# Hydrocephalus in a rat model of Meckel Gruber syndrome with a TMEM67 mutation

**DOI:** 10.1038/s41598-018-37620-5

**Published:** 2019-01-31

**Authors:** Joon W. Shim, Paul R. Territo, Stefanie Simpson, John C. Watson, Lei Jiang, Amanda A. Riley, Brian McCarthy, Scott Persohn, Daniel Fulkerson, Bonnie L. Blazer-Yost

**Affiliations:** 1 Department of Biology, Indiana University - Purdue University Indianapolis, Indianapolis, IN 46202 USA; 2 Department of Medicine, Boston University School of Medicine, Boston, MA 02118 USA; 3 Biomedical Engineering Program, Weisberg Division of Engineering, College of Information Technology and Engineering, Marshall University, Huntington, WV 25755 USA; 4 Department of Radiology and Imaging Sciences, Indiana University School of Medicine, Indianapolis, IN 46202 USA; 5 Department of Neurological Surgery, Indiana University School of Medicine, Indianapolis, IN 46202 USA

## Abstract

Transmembrane protein 67 (TMEM67) is mutated in Meckel Gruber Syndrome type 3 (MKS3) resulting in a pleiotropic phenotype with hydrocephalus and renal cystic disease in both humans and rodent models. The precise pathogenic mechanisms remain undetermined. Herein it is reported for the first time that a point mutation of TMEM67 leads to a gene dose-dependent hydrocephalic phenotype in the Wistar polycystic kidney (Wpk) rat. Animals with TMEM67 heterozygous mutations manifest slowly progressing hydrocephalus, observed during the postnatal period and continuing into adulthood. These animals have no overt renal phenotype. The TMEM67 homozygous mutant rats have severe ventriculomegaly as well as severe polycystic kidney disease and die during the neonatal period. Protein localization in choroid plexus epithelial cells indicates that aquaporin 1 and claudin-1 both remain normally polarized in all genotypes. The choroid plexus epithelial cells may have selectively enhanced permeability as evidenced by increased Na^+^, K^+^ and Cl^−^ in the cerebrospinal fluid of the severely hydrocephalic animals. Collectively, these results suggest that TMEM67 is required for the regulation of choroid plexus epithelial cell fluid and electrolyte homeostasis. The Wpk rat model, orthologous to human MKS3, provides a unique platform to study the development of both severe and mild hydrocephalus.

## Introduction

Hydrocephalus may be congenital or develop as a consequence of trauma, infection, venous occlusion, tumors, or intracranial hemorrhage resulting in cerebrospinal fluid (CSF) overproduction, malabsorption, or mechanical blockage of flow. Hydrocephalus is observed in approximately 1 in 1000 births but may occur at any age including a poorly understood “normal pressure hydrocephalus” found in the elderly^1^. Depending on severity and duration, hydrocephalus may cause developmental delay, progressive neurological decline, blindness, impaired motor function, urinary incontinence, dementia, or death.

The classic but oversimplified definition of hydrocephalus describes two types, communicating and obstructive^2,3^. In communicating hydrocephalus, the connections between the ventricles are open, but there is excess fluid resulting from an imbalance of CSF secretion, movement and/or absorption^4–6^. Obstructive hydrocephalus results from a mechanical blockage of the circulation of CSF^7^. Hydrocephalus is commonly treated by surgical placement of a shunt to divert the CSF^7^ or an endoscopic third ventriculostomy (ETV) to create a channel bypassing a site of obstruction. More recently a growing number of infants are treated with an ETV and choroid plexus cauterization^8^.

Ciliopathies are a spectrum of genetic disorders where proteins found in the primary cilia are mutated. The primary cilium is a cellular appendage on the apical membrane of polarized cells that functions as a mechano- or chemo-receptor and also plays a role in the formation of left-right asymmetry during development^9^. The most common of the ciliopathies is polycystic kidney disease (PKD). Other ciliopathies such as nephronophthisis (NPHP), Joubert syndrome (JS), Meckel-Gruber Syndrome (MKS), and Bardet Biedl Syndrome (BBS), all have central nervous system defects^10,11^. The cilia in these diseases show abnormalities, including truncation, elongation or diminished numbers. How different ciliary phenotypes result in functional defects of the nervous system remains poorly characterized.

The Wpk rat, carrying a single point mutation in the transmembrane protein 67 (TMEM67), is a genetic model of hydrocephalus and PKD that is orthologous to human MKS type 3 (MKS3)^12,13^. TMEM67 is one of a complex of co-localized proteins that, when mutated, cause MKS or JS^14–17^. In renal tissue, and mouse embryonic fibroblasts, TMEM67 localizes in the plasma membrane and in the area of the ciliary transition zone, a region between the basal body and axoneme. TMEM67 has been characterized as one of the proteins that serve as a “filter” controlling protein movement into the primary cilium^14,18^. In Wpk rats, the TMEM67^−/−^ homozygous animals have renal cystic disease with severe hydrocephalus and survive for approximately three weeks^12^. The heterozygous animals have no renal phenotype and breed normally in the first year.

The current studies are designed to compare the genotype and phenotype of Wpk rats and extend the characterization of cerebral abnormalities. The hydrocephalus is a communicating form of the disease as evidenced by an open cerebral aqueduct of Silvius in both the homozygous and heterozygous genotypes. During post-natal development of the homozygous hydrocephalic animals, the disease becomes severe with bilateral fusion of lateral ventricles. While the anatomical structure and polarity of the choroid plexus epithelial cells remain intact in the homozygous pups, there is an altered barrier function and electrolyte transport across the choroid plexus epithelia. The heterozygous (TMEM67^+/−^) rats have midline malformations and mild hydrocephalus that does not appear to impair normal physiological functions until after the first year of life. The latter is, to our knowledge, the first description of an animal genetic model of slowly progressing hydrocephalus. These models are unique in that they avoid the necessity of intracranial injection of sclerotic or hemorrhagic agents to induce the disease. Both the heterozygous and homozygous rat models may be useful for detailed physiological, behavioral and pharmacological studies.

## Results

### Identification of the TMEM67/MKS3 heterozygous mutation using dCAPS markers

**Figure 1:**
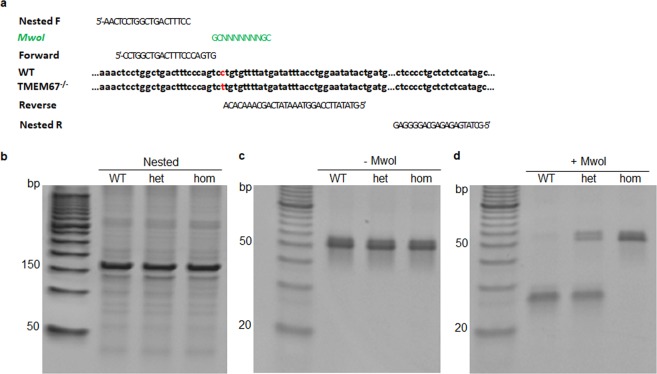
Characterization of the TMEM67 genotype in the Wpk rat. (**a**) Design of dCAPs genotyping approach. Primers for nested PCR are labeled Nested F and Nested R. MwoI indicates the sequence and location of the *MwoI* site created during PCR with the upstream and downstream dCAPs primers (labeled Forward and Reverse, respectively). The WT genomic sequence is labeled WT and the mutant sequence is labeled TMEM67^−/−^. The C → T mutation in the Wpk mutant is marked in red. (**b**) Polyacrylamide gel analysis of nested PCR products obtained from genomic DNA from (left to right) WT, heterozygous, or homozygous mutant rats. The expected product size was 157 bp. The left lane contains DNA length markers. Note that in the first lane to the left, a 50 bp DNA ladder (50–1350 bp) was used as a standard with 50 to 150 bp markers indicated (**c**) A polyacrylamide gel as in (**b**) except that the nested PCR products were amplified with the dCAPs primers (see **a**). The expected product size was 51 bp. (**d**) A polyacrylamide gel as in (**c**) except that the dCAPs PCR products were digested with MwoI prior to electrophoresis. MwoI digestion of the dCAPS product from the WT allele is expected to produce two unresolved bands migrating slightly faster than the 30 bp marker. MwoI is not expected to cleave the dCAPs product from the mutant allele. Note that in the first lane from the left of both gels (**c**,**d**), a 10 bp DNA ladder (10–150) was used as a standard with the 20 to 50 bp markers indicated.

The Wpk rat was previously shown to have a single C to T substitution within exon 12 of the TMEM67/MKS3 gene that converts a proline to a leucine in the polypeptide^13^. Although this mutation reduces the transcript level in kidney as determined by RNA gel blotting, there was little or no change in TMEM67/MKS3 RNA in brain tissues reported previously^13^, reducing the utility of RNA gel blotting as a genotyping tool for these studies. Moreover, to our knowledge, neither DNA blotting nor PCR methods for detecting the mutant allele had been previously developed. To overcome this limitation, we used a PCR-based method involving dCAPS markers^19^. We designed mismatched primers flanking the mutation to create a cleavage site for MwoI only in the wild type (WT) allele (Fig. 1a). However, amplification of rat genomic DNA with the dCAPS primers generated several PCR products in addition to the 51 bp target because of related sequences in rat genomic DNA. Therefore, nested PCR was used to first amplify a 157 bp region containing the target sequence (Fig. 1b), followed by a second amplification using the dCAPS primers (Fig. 1c). After MwoI digestion, the PCR product from WT animals generates two closely migrating fragments just below the 30 bp marker (Fig. 1d, TMEM67^+/+^; n = 98). In contrast, the product from homozygous mutant animals was uncleaved, migrating near the 50 bp marker (Fig. 1d, TMEM67^−/−^; n = 72). Heterozygotes were identified by the presence of the 51 bp product as well as the MwoI digestion products (Fig. 1d, TMEM67^+/−^; n = 234). The dCAPS genotyping correlated well with the cerebral phenotypes described below. This technique provides the ability to unambiguously characterize the heterozygous animals for the first time and determine potential cerebral changes that result from a diminished TMEM67 protein expression.

### Neonatal development of the Wpk model

**Figure 2:**
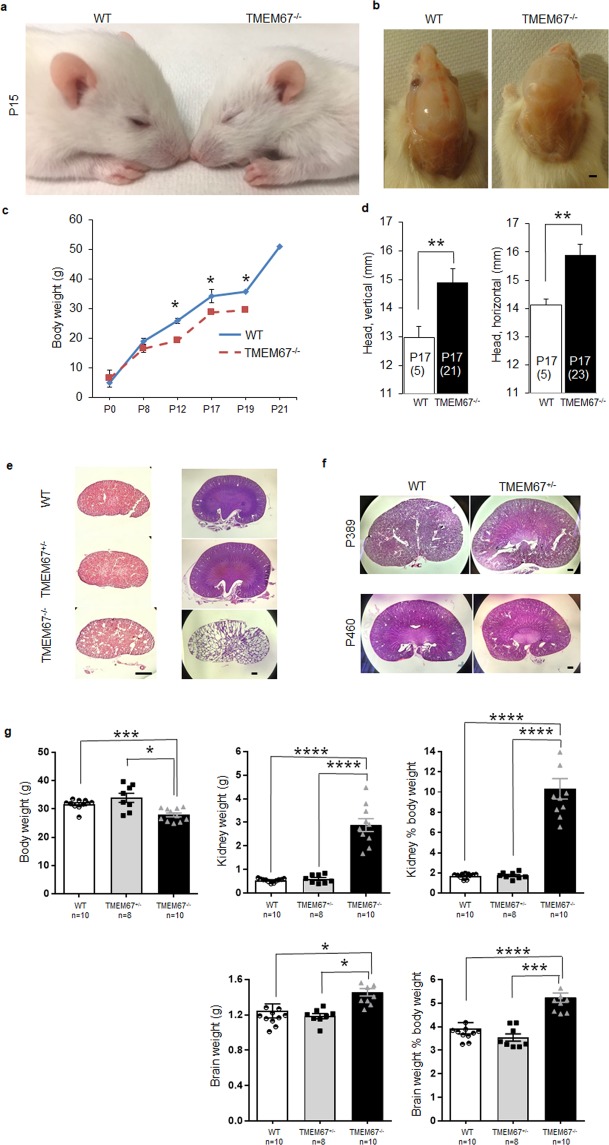
Correlation of phenotypic and genotypic characteristics in the Wpk rat. (**a**) A TMEM67^−/−^ Wpk rat displaying a dome-shaped head with reduced body size compared to a WT littermate at P15. (**b**) Typical cranial doming at P17 in the Wpk homozygous rat as compared with a sibling WT animal. (**c**) Comparison of body weights of WT and TMEM67^−/−^ rats from postnatal day 0 to 21 (P0-P21). The TMEM67^−/−^ mutants had a significantly reduced body weight at P12 (p = 0.012), P17 (p = 0.015), and P19 (p = 0.01) as compared to WT. The homozygous animals were humanely sacrificed at day 19. (**d**) Measurement of head sizes of WT and homozygous mutant rats at P17. Head sizes in the homozygous animals were significantly increased over the control animals when measured in vertical (palate to cranial cap) (p = 0.006) or horizontal (biparietal) orientation (p = 0.007). Numbers in parentheses at the base of the columns indicate the number of animals measured. (**e**) Representative renal phenotypes of the WT, TMEM67^+/−^ and TMEM67^−/−^ rats at P0 (hematoxylin stain) and P15 (hematoxylin and eosin stain). (**f**) Representative adult renal phenotypes of the WT and TMEM67^+/−^ and TMEM67^−/−^ rats at P389 and P460 (hematoxylin and eosin stain). (**g**) Body weight, kidney weight, brain weight, and related organ weight as a percentage of body weight. At the time of sacrifice (P15), body weight and organ weight were determined and the organ weight as a percentage of the total body weight was calculated. The numbers of animals analyzed are indicated on the graphs. Scale bars, 1 mm (**b**,**e**,**f**). Single (*) double (**) triple (***) and quadruple (****) asterisks denote p < 0.05, p < 0.01, p < 0.005, and p < 0.0001, respectively.

The hydrocephalus in the TMEM67^−/−^ homozygous animals is severe, resulting in cranial doming (Fig. 2a,b,d). At post-natal (P) day 17, the head dimensions of the homozygous animals were significantly increased over the control animals when measured in vertical (palate to cranial cap) or horizontal (biparietal) orientation (Fig. 2d). The homozygous pups had a significantly reduced body weight at P12, P17, and P19 as compared to WT and died postnatally prior to weaning age (Fig. 2a,c). The renal consequence of the TMEM67 mutation presented as severely cystic kidneys in the homozygous pups while both heterozygous and WT animals had normal kidneys (Fig. 2e,g). These results are consistent with the previous reports on the non-cystic and cystic Wpk rat model^12,13,20^ but represent the first depiction of a phenotype in heterozygous animals. As the animals age to adulthood, the renal phenotype of the heterozygous animals remains normal (Fig. 2f). Unlike whole body and kidney, heart weight did not show a significant difference in the homozygous animals as compared with WT controls at P17 and the hematocrits were also normal (data not shown).

To substantiate the initial findings and to further characterize the heterozygous animals, a second cohort was examined at post-natal day 15 (Fig. 2e,g). As in the first cohort, the homozygous animals showed a statistically significant decrease in body weight at P15. There was no difference between the WT and heterozygous animals. The brains and kidneys of these animals were also collected and weighed. Whether the kidney weights or the brain weights were expressed as organ weight or as organ weight as a percentage of body weight, the homozygous pups were statistically increased over the other two genotypes. The WT and heterozygous animals were the same in all parameters (Fig. 2g).

### Cerebral phenotype of the neonatal Wpk rats

**Figure 3:**
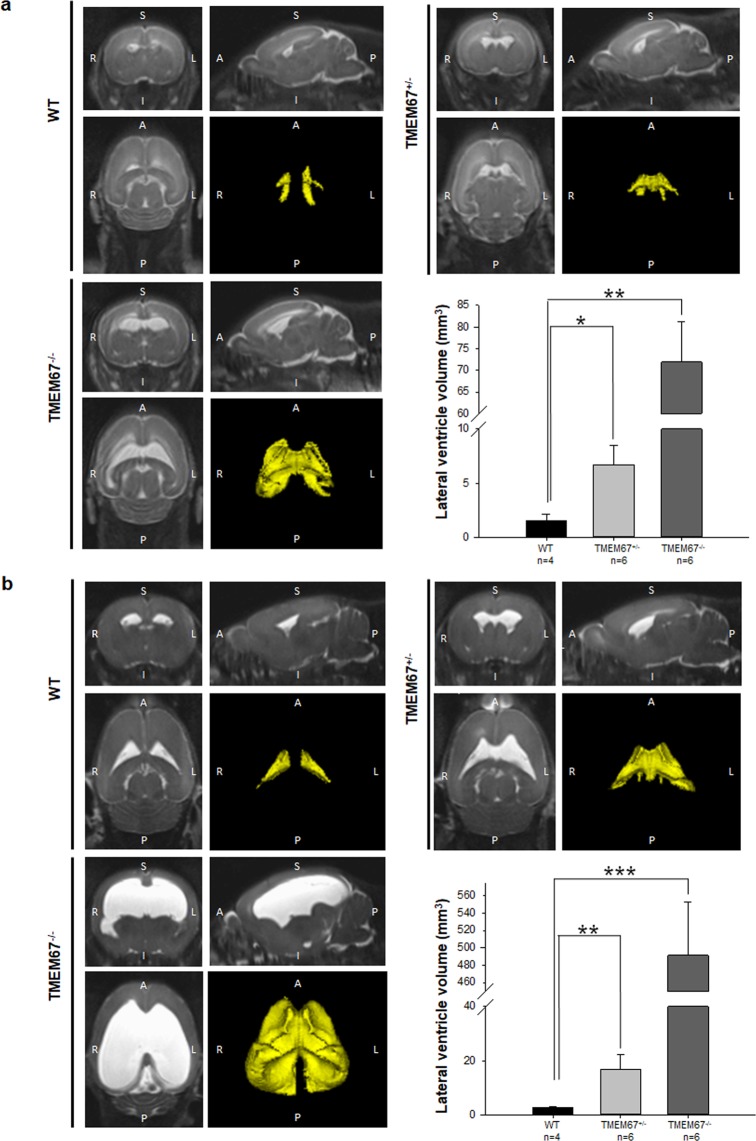
Magnetic Resonance Imaging (MRI) of the three genotypes of the pre-weaning Wpk rat model with TMEM67 mutations. (**a**) Head scan of Wpk rats at P7–8 in coronal, sagittal, and transverse orientation with 3 dimensional reconstructions of lateral ventricle (LV; yellow). (**b**) Head scan of Wpk rats at P17–18 in coronal, sagittal, and transverse orientation with 3 dimensional reconstructions of the LV (yellow). A bar graph summarizing the quantitative data of the lateral ventricles per genotype is shown in the lower right corner. Note that the heterozygous animals (middle) show intermediate size in LV volume. n = 4 to 6 per genotype. Asterisks denote *p < 0.05, **p < 0.01, and ***p < 0.001, respectively.

To determine and compare ventricular volumes in the Wpk rats of all three genotypes, the pups were scanned using MRI at two postnatal time points. At P7–8, volumes of the lateral ventricle for WT, heterozygotes, and homozygotes were 1.5 ± 0.6, 6.7 ± 1.8 and 71.9 ± 9.2 μl. After a further 10 days of maturation (P17–18), the respective lateral ventricle sizes were 2.6 ± 0.3, 16.7 ± 5.4 and 491.2 ± 61.9 μl, respectively (Fig. 3). Interestingly, the fold change in lateral ventricular volume during neonatal aging (P7–8 to P17–18) for WT (1.7×), heterozygous (2.5×) and homozygous (6.8×) animals was gene dosage dependent and significantly different between genotypes. Measurements in these young rats displayed the same order of magnitude as a previous report on basal cistern kaolin-injected hydrocephalus, in which ventricular volume was quantified in adult female rats^21^.

### Cerebral characteristics in the TMEM67^+/−^ rats

**Figure 4:**
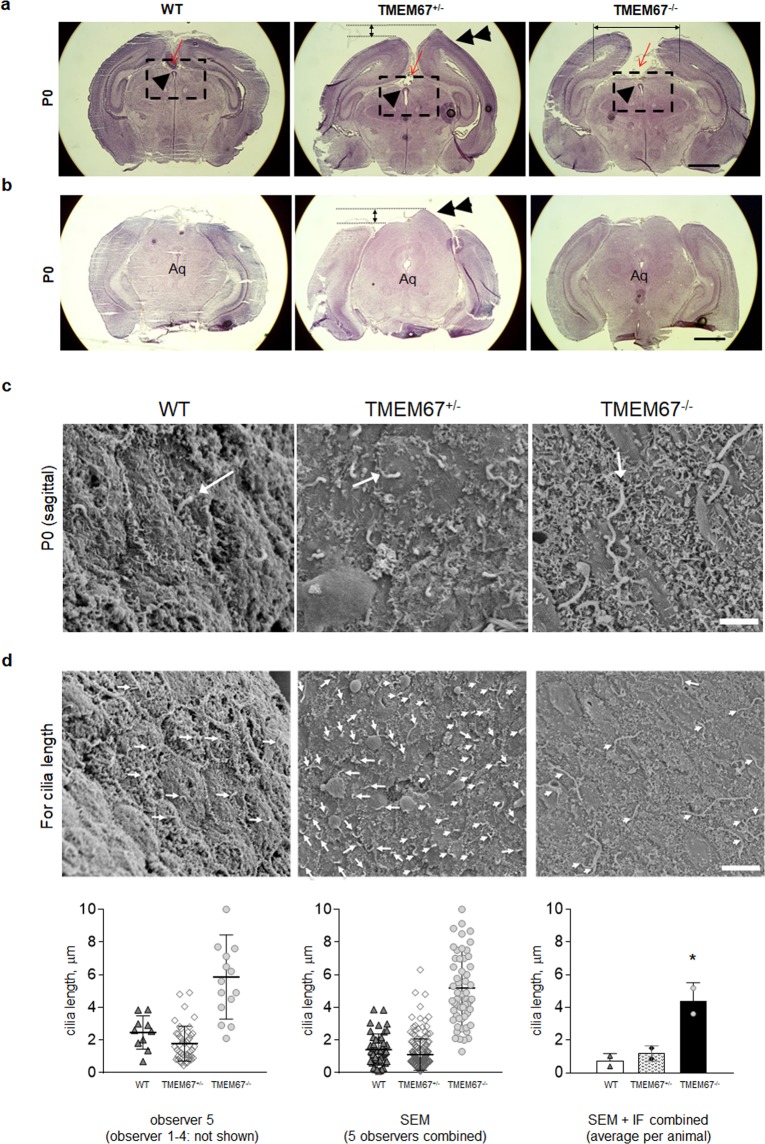
Midline malformation and ciliary phenotype at birth in the TMEM67^+/−^ rats. (**a**) Twenty μm (thickness) sections of dorsal cerebral cortex along the midline in the hindbrain. Note that a sac-like protrusion (double arrowheads) is found in the left hemisphere of TMEM67^+/−^. At P0 a wide gap is detected between two hemispheres in TMEM67^−/−^ as compared to WT. Red arrows indicate usual location of pineal gland. In TMEM67^−/−^, a bloody membrane was observed. The single arrowhead indicates the subcommissural organ (SCO). (**b**) A sac-like protrusion in the occipital lobe of TMEM67^+/−^ (double arrowheads) as compared with WT at P0. Aq denotes aqueduct. Note that aqueduct is open in all genotypes at birth. n = 3 per genotype (a representative section per genotype is shown in a–b; hematoxylin and eosin stain) (**c**) Scanning electron micrographs displaying primary cilia on the striatal side of the ependyma in WT, TMEM67^+/−^ and TMEM67^−/−^ at P0. (**d**) 5 blinded observers measured cilia length as indicated with arrows. The scattered plot of the representative measurement by an observer (left), the scattered plot of the combined measurement by five observers (middle), and the bar graph showing the combined measurement of the SEM and IF using anti-Arl13b staining of cilia (not shown) are presented in the lower panel. Asterisk denotes *p < 0.05 (as compared to WT and TMEM67^+/−^, respectively). Two animals per genotype. Scale bars, 1 mm (**a**,**b**), 2 μm (**c**), and 5 μm (**d**).

A sac-like protrusion was found during sectioning of early postnatal brains of the heterozygous animals suggesting an abnormality in the parietal to occipital lobe. In a coordinate, relatively caudal to the center of the brain where the pineal gland was seen, the heterozygous animals showed an elevated left hemisphere (V2MM; secondary visual cortex mediomedial area) as compared to that of sibling WT animals at P0. Interestingly, homozygous rats did not show the asymmetry at this coordinate but, rather, the presence of a gap between two hemispheres with remnants of thrombotic membrane suggestive of venous sinus thrombosis (Fig. 4a). In a more caudal coordinate where the aqueduct was present, the brains from the heterozygous animals retained the elevated height in the left hemisphere (ECIC; external cortex of the inferior colliculus) as compared to that of WT at P0 (Fig. 4b).

Primary cilia are involved in tissue patterning during development; therefore, to determine potential effects of ciliary defects in the brain, the ependyma was examined using scanning electron microscopy of the brain in a sagittal orientation. At birth (P0), mono-cilia were detected in WT and heterozygous animals with occasional presence of ciliary tuft in the ventricular surface. In the homozygous ependyma, however, the mono-cilia (primary cilia) appears markedly longer than those of the heterozygous or WT controls (Fig. 4c). In agreement with a previous report^12^, at latter time points (P8 and thereafter), the ventricular surface was covered with tufts of cilia, which were very similar in WT and homozygous rats (data not shown). To quantify the ciliary phenotype of the ependyma revealed in the SEM, the cilia length was measured by 5 independent, blinded observers who were naïve to the experimental protocol. A representative count by one observer using SEM images and all the counts by five observers combined are shown in the first and second scattered plots of Fig. 4d. The results, then, were validated in the separate additional experiment using immunofluorescent images of anti-Arl13b stained cilia from another set of experiment (micrographs not shown). Both sets of two independent experiments using SEM and immunostaining indicated that the cilia of TMEM67^−/−^ were significantly longer than other genotypes as shown in the bar graphs of Fig. 4d.

**Figure 5:**
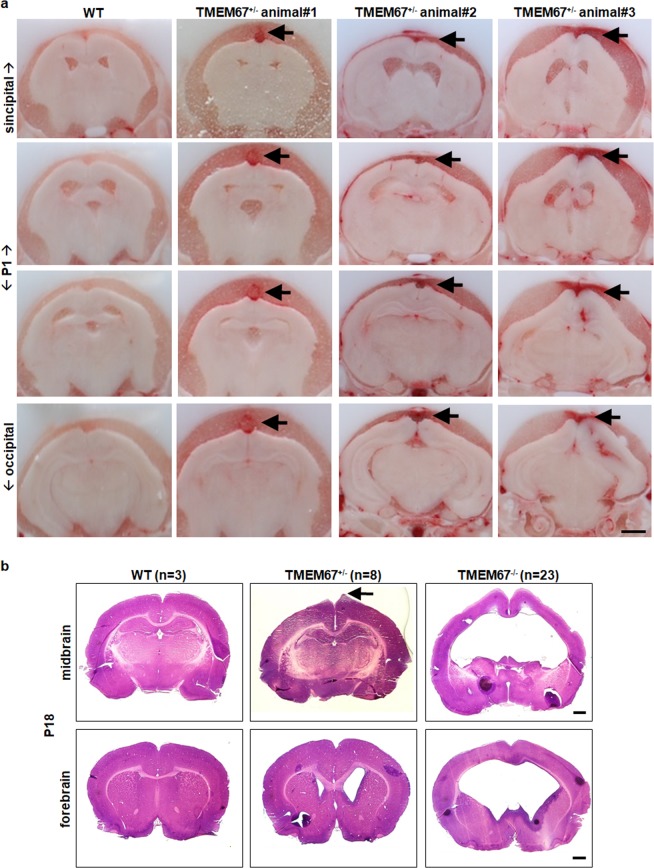
Hemorrhage at birth and neonatal ventriculomegaly. (**a**) Hemorrhage in the TMEM67 heterozygous mutant brains at birth. The left panel demonstrates a representative serial section of a WT (TMEM67^+/+^) rat from the forebrain (sincipital) to hindbrain (occipital) at postnatal day 1 (P1). The subsequent three panels show three different animals with TMEM67^+/−^ alleles exhibiting hemorrhage (solid arrows) adjacent to the venous sinus within extra-axial space adjacent to the subarachnoid space at P1. Similar results were obtained for n = 3 animals per genotype. (**b**) Representative cerebral phenotype of the TMEM67^+/+^, TMEM67^+/−^, and TMEM67^−/−^ animals at P18, respectively. Note that mild ventriculomegaly was observed in the TMEM67^+/−^ (n = 8; 4 left enlarged, 2 right enlarged, 2 both sides) while bilateral dilation with fusion of lateral ventricles was seen in the TMEM67^−/−^ rats (n = 23). Arrow indicates a protrusion on the dorsomedial surface of the cerebral cortex in the TMEM67^+/−^ rat. Scale bars, 1 mm (**a**,**b**).

Because the midline protrusion in the heterozygous animals was not visible on the external surface of the skull, we assessed the forebrain part of the head including extra-axial space (EAS) that subsumes subdural and subarachnoid space. Strikingly, formation of hemorrhage in the dorsomedial surface within the EAS along the midline was detected in the heterozygous animals with an increase in size in dorsomedial EAS as compared to that of WT. At P1, the external hemorrhage in the EAS appeared as a bilateral protrusion with respect to the midline in the forebrain (Fig. 5a, solid arrow). At P18, there was no residual indication of hemorrhage but a protrusion similar to that observed in Fig. 4a was observed in the brains of these animals (Fig. 5b, solid arrow).

The ventricular enlargement in the homozygous animals became progressively worse with age, severely compromising other brain tissue by P18, the age when the affected animals show signs of terminal disease and were sacrificed for humane reasons. The serial sections confirmed ventriculomegaly with fusion of both ventricles (n = 23), whereas the heterozygous animals exhibited mild enlargement of the lateral ventricle (n = 8) (Fig. 5b). The asymmetric lateral ventricle and the midline protrusion in the heterozygous rat were observed starting at P8 until around P360 (n = 7).

### Communication of CSF in the TMEM67^−/−^ brain

**Figure 6:**
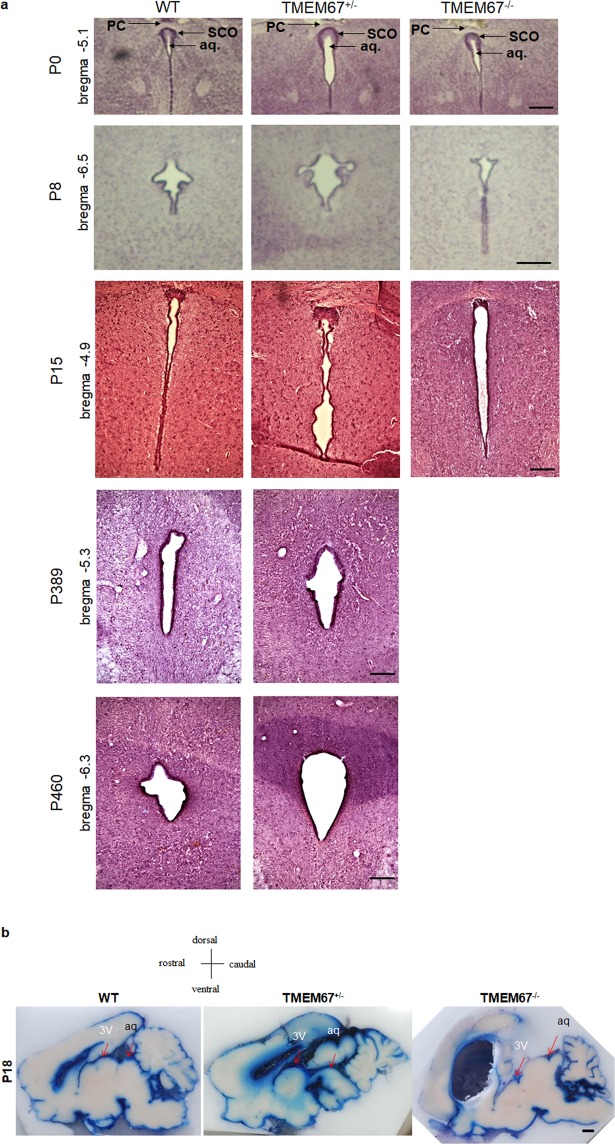
Communicating hydrocephalus. (**a**) Representative depictions of the aqueducts of the TMEM67^+/+^, TMEM67^+/−^, and TMEM67^−/−^ rats at P0, P8 and P15. Note that the aqueduct is open (arrow with “aq”) in TMEM67^−/−^. Approximate coordinates from the bregma are indicated based on the rat brain atlas. The images are representative of n = 3 animals of each genotype and age. (**b**) Sagittal sections injected with Evans Blue to determine continuity (communication) or discontinuity (obstruction) of the CSF flow. Dye leakage along the CSF circulation including lateral ventricle, third ventricle, aqueduct and fourth ventricle was visualized by the cisterna magna injection of Evans blue in TMEM67^+/+^, TMEM67^+/−^, and TMEM67^−/−^ rats at P18. 3 V and aq. denote third ventricle and aqueduct, respectively. Scale bars, 250 μm (**a**) and 1 mm (**b**). The images are representative of n = 3 at each genotype.

To determine the type of hydrocephalus occurring in the Wpk rat, we examined the caudal CSF space and circumventricular organ at the midline. The SCO did not show a significant change at P0 (Fig. 6a). The cerebral aqueduct remained open in all genotypes during neonatal development and into adulthood (Fig. 6a).To further substantiate the finding of communicating hydrocephalus indicated by the open aqueducts, a dye extravasation study was performed on all genotypes by injecting Evans blue in the cisterna magna of Wpk rats at P18. Blue dye leakage suggests that there was no obstruction along the CSF circulation including aqueduct and ventricles in either the homozygous or heterozygous rats (Fig. 6b). However, the Evans blue trait along the aqueduct and cistern of the homozygous brains was less intense than that of heterozygous and WT brains. Taken together, the Evans blue dye injection through the cisterna magna visualized in sagittal orientation supports the histological observation in coronal serial sections that communicating hydrocephalus is present in the TMEM67 homozygous mutant rats.

### Cerebral phenotype in adult TMEM67^+/−^ rats

**Figure 7:**
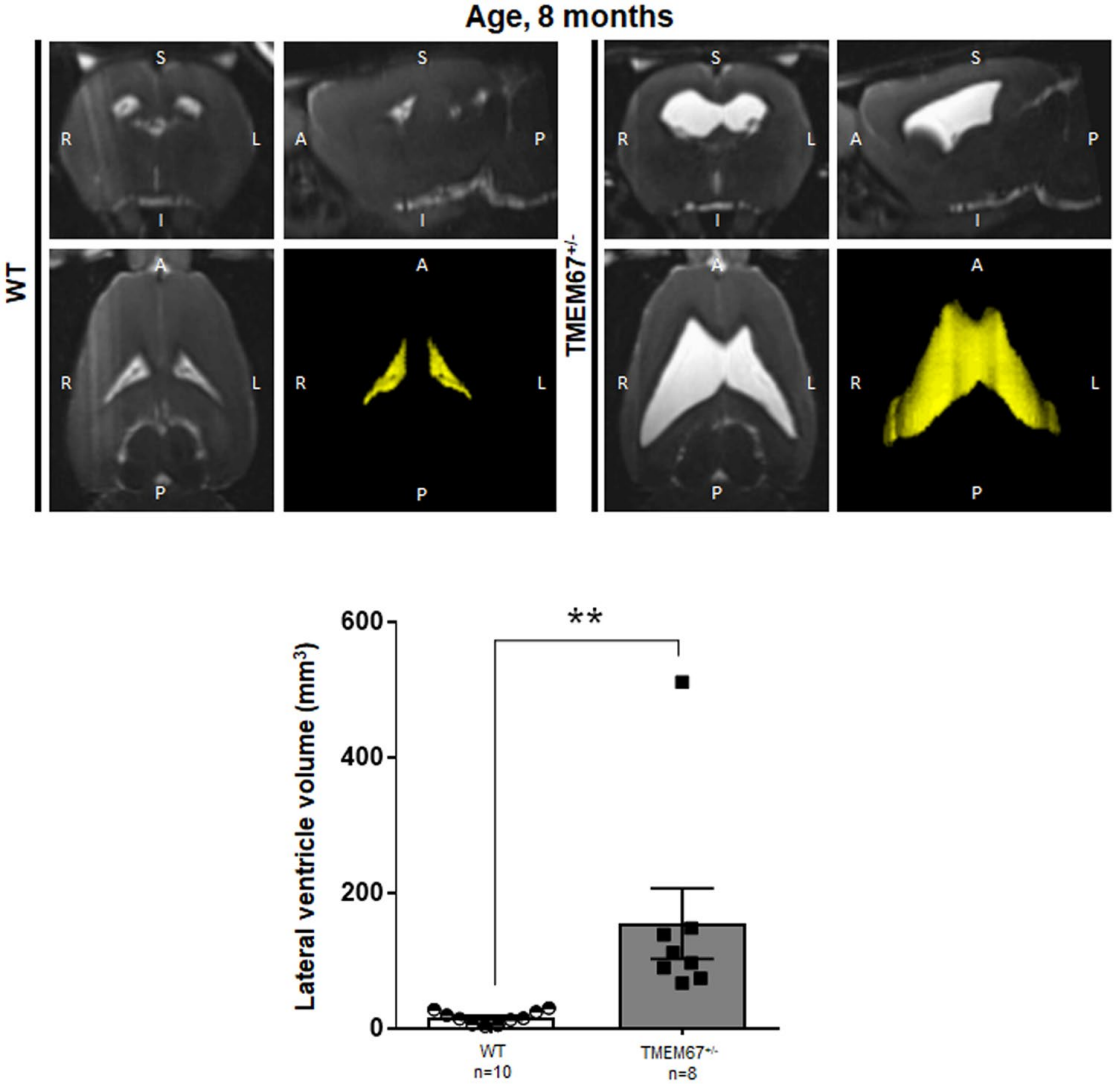
Magnetic Resonance Imaging (MRI) of the WT and heterozygous (TMEM67^+/−^) Wpk rats at 8 months of age. Head scan of Wpk rats at P240 ± 3 days of age in coronal, sagittal, and transverse orientation with 3 dimensional reconstructions of lateral ventricle (LV; yellow). A bar graph with scattered dots summarizing the quantitative data of the lateral ventricles per genotype is shown in the third panel. Numbers of animals scanned are indicated at the bottom of each column. Double asterisks (**) denote p < 0.01.

To determine if the mild hydrocephalus that is present in pre-weaning animals continues through adulthood, brain scans and analysis of MRIs were performed to compare WT and heterozygous animals at 8 months of age. There is a statistically significant difference in the size of lateral ventricles between the WT and heterozygous animals (Fig. 7). Interestingly, the asymmetry of the ventriculomegaly in the heterozygous animals continues into adulthood. Eight of eight (100%) of the heterozygous (TMEM67^+/−^) rats developed ventriculomegaly at age 8 months as compared to the age-matched WT (n = 10). All animals were included in the analysis and there was no overlap in ventricular volume between the WT and heterozygous animals (Fig. 7).

### Choroid plexus epithelium and barrier function

**Figure 8:**
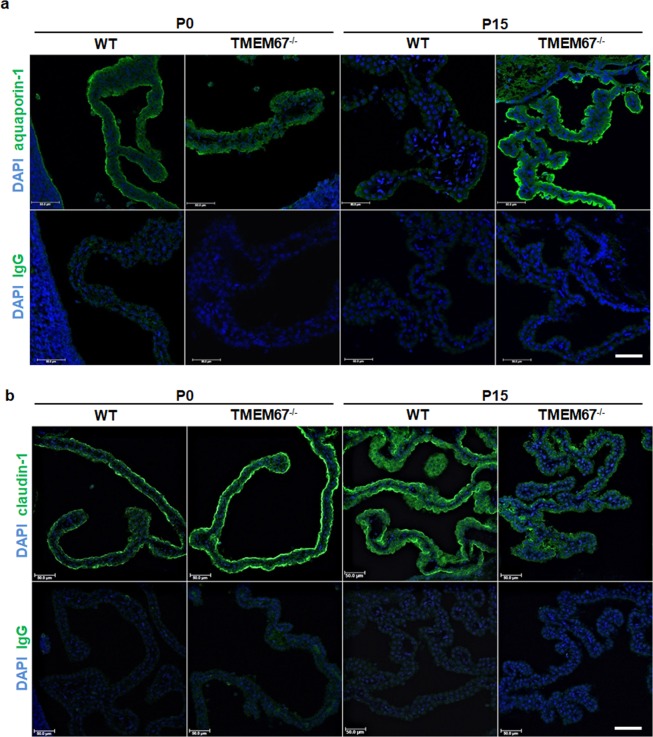
Immunofluorescence of membrane proteins in the Wpk choroid plexus. Brain tissue was sectioned at 20 µm and incubated with primary antibody for the water channel, aquaporin 1, or the tight junction protein, claudin-1 (1:100 antibody dilutions). Protein expression is shown in WT and hydrocephalic rats at birth (P0) and P15. DAPI staining allows for visualization of nuclei. Negative (−) controls were incubated with secondary antibodies (1:1000 dilution, AlexaFluor goat anti-rabbit IgG). Images were taken on a Leica TCS SP8 (upright high-speed multiphoton and confocal imaging system) at 20x magnification. Scale bars, 50 µm. These images represent an n of at least 3 for each genotype and age.

The choroid plexus epithelial cells are responsible for the nature of the blood-choroid plexus barrier. In addition, polarization of membrane proteins is necessary for vectorial osmolyte transport. To further characterize these epithelial cells in the disease state, the polarization of relevant membrane proteins was examined. Aquaporin 1, a water channel present in the choroid plexus was localized primarily to the apical plasma membrane in plexuses derived from new-born WT and homozygous pups. This localization was maintained in the homozygous animals at 15 days of life (Fig. 8a).

Claudin 1, considered a barrier claudin, is a tight junction protein important for maintenance of transepithelial permeability^22,23^. Claudin 1 staining is similar in the newly born WT and homozygous pups but decreased in intensity after 15 days in the homozygous animals (Fig. 8b). However, despite the decrease in intensity, the claudin-1 polarity to the apical junctions is maintained in the older pups.

Taken together, the localization studies indicate that the cells are viable and that a major transport protein as well as a junctional complex protein are appropriately polarized, albeit, with possible changes in quantitative expression levels.

**Figure 9:**
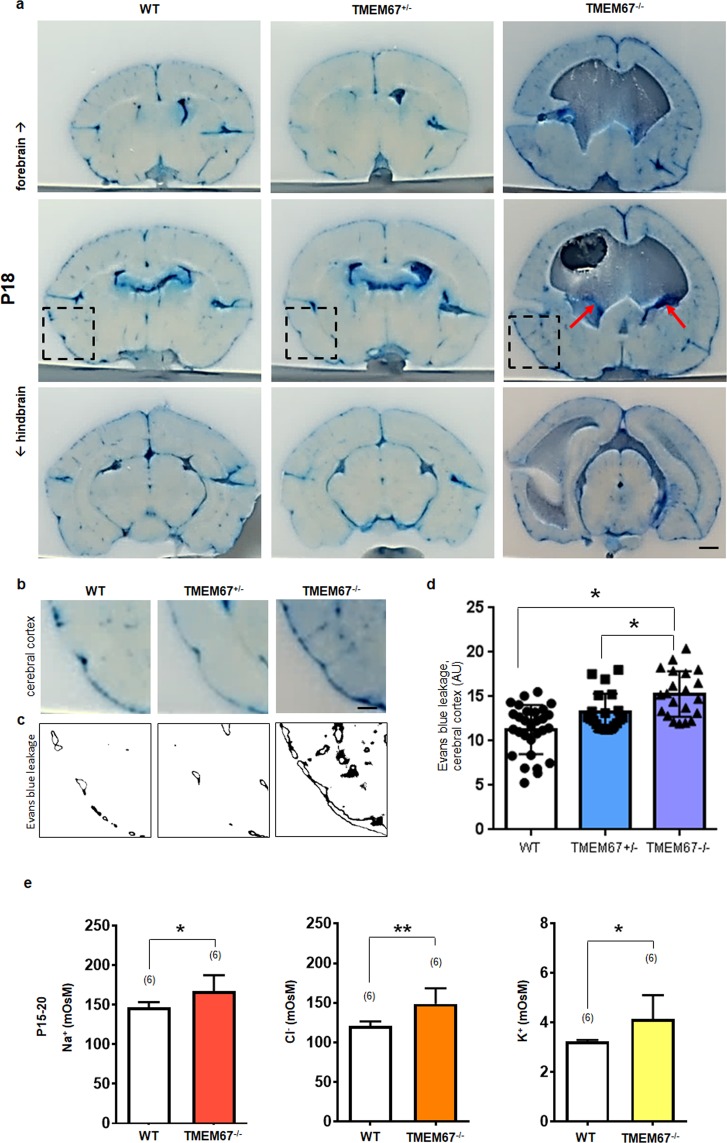
Evans blue leakage in the brain after intracardial injection of the dye. (**a**) Representative coronal sections displaying Evans blue leakage in all three genotypes of the Wpk rat. Arrows indicate blue dye leakage in the choroid plexus in the lateral ventricles of a TMEM67^−/−^ brain. The images are representative of n = 3 at each genotype. (**b**) Magnified image of Evans blue leakage in the cerebral cortex, from dashed rectangles shown in a. (**c**) Binary image of Evans blue positive pixels quantified in the cerebral cortex obtained from b. (**d**) Box plot exhibiting statistical analysis of Evans blue leakage along the vasculature of the cerebral cortex as exemplified on images in c. Note that Evans blue binds to albumin. Evans blue injection indicates a trend towards a dose-dependent increase of the dye extravasation in the cerebral vessels. Serial sections (n = 21–29) from three animals per genotype were used. AU, arbitrary unit. Asterisks denote *p < 0.05. Scale bars, 10 μm (**a**) and 1 mm (**b**). (**e**) Ionic composition of the CSF from TMEM67 rats: Bar graphs demonstrating an osmotic concentration in milliosmolar (mOsM) of sodium (Na^+^), chloride (Cl^−^), and potassium (K^+^) in the CSF of WT and TMEM67^−/−^ rats, postnatal day 15–20. Each bar represents 6 determinations. In the case of the homozygous animals, each determination was from the CSF of a single individual. For the normal animals, CSF from 4–6 individuals was pooled to obtain sufficient material for each determination. Statistical significance from the left to the right. P = 0.0103 (CSF Na^+^); P = 0.0013 (CSF Cl^−^); P = 0.041 (CSF K^+^) by unpaired t test. Single and double asterisks denote p < 0.05 and p < 0.01, respectively. Scale bars, 1 mm (**a**) and 500 μm (**b**).

The vascular permeability of the TMEM67 mutant brains was assayed to address changes in barrier function in animals with hydrocephalus^24,25^. Intracardial injection of Evans blue demonstrated that blue dye extravasation was significantly increased in brains from homozygous as compared to WT animals. Interestingly, we noted that Evans blue leakage was detected in the choroid plexus of the TMEM67^−/−^ rats (arrows, Fig. 9a). To examine leakiness of the vasculature in the cerebral cortex, we administered intracardial Evans blue dissolved in saline. The area of Evans blue dye extravasation excluding the ventricular surface was measured which eliminated the confounding effect of dye leakage in the ventricles including the choroid plexus. Dye leakage in the cerebral cortex of the homozygous rats excluding ventricles was significantly elevated (p = 0.002; Kruskal-Wallis with Dunnett’s post hoc tests) as compared to WT animals (Fig. 9a–d).

Minor changes in vascular leakage and/or a change in the epithelial permeability of the blood-CSF barrier might be expected to have functional consequences manifested in the composition of the CSF. Of the major electrolytes found in the CSF of late-stage hydrocephalic rats compared to control animals. Na^+^, K^+^ and Cl^−^ are all statistically elevated in the hydrocephalic animals (Fig. 9e).

## Discussion

Hydrocephalus is a serious disease that can occur at any age and can arise from a multitude of causes. Regardless of cause, the main treatment is surgical intervention to divert excess CSF to other areas of the body and/or ablation of the choroid plexus to decrease CSF production. Development of pharmacological interventions has been hampered, in part, by a lack of physiologically relevant animal models, particularly those that demonstrate a more slowly progressing form of the disease and live long enough to allow a reasonable course of drug treatment.

The TMEM67 homozygous rat is an orthologous model of MKS3 and demonstrates many of the characteristics of the human disease including severe polycystic kidney disease and brain abnormalities^12,13,20^. Like the homozygous rats, the human patients with MKS die in the perinatal or neonatal period^10^. In the course of the current studies, we made the fortuitous observation that the heterozygous breeders had a mild form of hydrocephalus that appears to adversely affect the animals at approximately one year of age when they begin to show moderate signs of stress like failure to groom. Because of the difficulties in genotyping animals with a single nucleotide mutation, previous studies failed to distinguish WT from heterozygous animals and breeders were classified as heterozygous when they produced off-spring with neonatal hydrocephalus and grossly enlarged kidneys^12,20^. Accurate genotyping of the heterozygous Wpk animals by combining a nested PCR and dCAPS approach allowed us to genotype the animals shortly after birth and provided the ability to characterize the heterozygous animals in the neonatal period. Particularly important in this regard is the ability to distinguish differences in the ventricular volumes of all three genotypes as early as post-natal day 7–8 by MRI.

Due to both the severe hydrocephalus and cystic renal disease, homozygous pups succumb to the disease in the first few weeks of life. There is a statistically significant decrease in weight gain as early as post-natal day 12. At post-natal day 15, the kidney and brain weights of the homozygous animals are significantly increased while, interestingly, the heterozygous animals are indistinguishable from WT in both parameters. In the homozygous animals, it has been reported that the disease results in fatality by 4–6 weeks^20^. In the current studies, these animals were humanely sacrificed at or before post-natal day 18. Of interest for future studies is the finding that, while heterozygous animals maintain a hydrocephalic state through adulthood, there is no evidence of cystic kidney disease in the adult animals.

Both genotypes appear to express a communicating form of hydrocephalus. This is indicated by the open cerebral aqueduct on Silvius in both the neonatal and adult animals. In addition, Evan’s Blue injection into the cisterna magna at P18 suggests that there was no obstruction along the CSF circulation including aqueduct and ventricles in either the homozygous or heterozygous rats. These results are consistent with a previous report^26^, using a mouse model of a different ciliopathy, BBS, where the authors also found a communicating form of hydrocephalus after Evan’s Blue injection.

In interpreting the MRI results of the adult animals, it is important to note that Wistar rats have a high degree of spontaneous ventriculomegaly. In a study by Tu *et al*., the incidence of enlarged ventricles was 19%^27^. In the current study, no animals were excluded from analysis and no increased ventricular size was detected in any of the WT adults (n = 10) that were analyzed by MRI. In addition, there was no overlap in the size of the lateral ventricles when comparing adult WT and heterozygous animals of the same age.

The TMEM67 gene appears to be related not only to ventricular size but also to regulation of cilia length and midline morphogenesis. An elongated ciliary phenotype has been reported in primary cilia of extra-cerebral tissues from patients with Meckel syndrome^11^ and in Wpk rats^28^. Studies of at least two other *in vivo* ciliopathy models of polycystic kidney disease (jck and cpk mice), have also reported the unusually long primary cilia in the kidney and bile duct^29,30^. In the ventricles, primary cilia are virtually impossible to measure after P0 because the animals develop tufted motile cilia which obscure the primary cilia within a day after birth. Previously published studies have shown that there is no difference in the tufted cilia between the WT and homozygous animals^12^. However, the renal cilia length has been published for the WT and homozygous rats^28^. In the kidney, one does not have the confounding variable of tufted, motile cilia. Tammachote and colleagues reported a statistically significant increase in the number of cilia with lengths greater than 3 μM in the homozygous animals. It should be noted that in the previous report^28^, genotyping was not done so the unaffected animals would be a mixture of WT and heterozygous pups. The current results are consistent with the previous studies. In the homozygous animals, the primary cilia are statistically longer than those of the heterozygous or WT animals (1 or < 1 μm) and are also longer than 3 μm. An entirely new finding is that the cilia from WT and heterozygous animals are not statistically different at birth.

Hemorrhage in the brain is a primary causal factor in the development of communicating hydrocephalus^25,31–33^. Dorsomedial hemorrhage seen at P1 in the TMEM67 heterozygous rat and appears to be linked to midline malformation in the current study. Heterozygous pups (n = 3) showed dorsomedial hemorrhage in the extra-axial space adjacent to SAS. There was no evidence of hemorrhage after P7 and, interestingly, there was also no evidence of hemorrhage in the homozygous animals. The role of hemorrhage and its possible association with mild hydrocephalus needs further investigation.

One potential cause of aberrant fluid homeostasis in ciliopathies is the mislocalization of polarized proteins in barrier epithelial cells responsible for electrolyte and fluid movement^34,35^. However, we find no evidence of mislocalization in the current studies. The choroid plexus water channel aquaporin 1 shows a predominately apical expression in both the WT and homozygous animals at birth and this polarity is maintained at P15. Likewise, claudin-1, a junctional protein important for maintenance of epithelial barrier function^23^ shows an apical distribution, localized to the apical junctional complexes in both the WT and homozygous pups at birth. At day 15, the claudin-1 is still localized predominately on the apical side of the epithelia of the homozygous animals, albeit with a decreased intensity.

Thus, a junctional protein important for epithelial cell polarization, as well as a transport protein with a defined polarity in choroid plexus epithelia, maintain normal distributions. Despite the maintenance of normal cellular polarity, the Evan’s Blue studies indicate an enhanced dye leakage across the choroid plexus. Taken together these findings suggest a controlled increase in transepithelial permeability.

The endothelial cells of the choroid plexus have been characterized as a fenestrated capillary. Therefore, they form an area of the vasculature that is leaky to small molecular weight compounds. Under normal conditions it is the epithelial cells of the choroid plexus, a high resistance epithelial monolayer, that form the blood-CSF barrier. Under normal conditions, polarized electrolyte transport across the epithelium results in CSF that contains a higher concentration of Cl^−^ and a lower concentration of K^+^ than plasma^4,36^. Na^+^ concentrations are approximately equal in these two extracellular fluids. It follows, therefore, that if the development of hydrocephalus in the rat model is due to a non-specific increase in permeability across the epithelial cell barrier, the resulting CSF would have a composition more similar to a plasma filtrate. In this scenario, the Cl^−^ concentration would decrease and the K^+^ concentration increases while the Na^+^ concentration would be relatively unchanged. Surprisingly, this is not the case in the severely hydrocephalic animals where all three electrolytes are higher in the CSF compared to WT. These results further substantiate a controlled and specific increase in electrolyte secretion across the choroid plexus epithelial cells and into the CSF.

From these results, the development of the hydrocephalus could be predicted to be due to the increased osmotic gradient, which would cause fluid accumulation in the CSF. Substantial further investigation is necessary to determine which transporters may be primary in this altered transepithelial transport. However, it is interesting to note that renal cystic development in ciliopathies has been shown to be primarily due to an aberrant regulation of transepithelial Cl^−^ transport^37,38^. Since the transporters responsible for maintaining of K^+^ and Cl^−^ gradients can be modulated by a variety factors including inflammatory cytokines, pressure, osmolarity, hormones, neuromodulators and intracellular signaling components, these proteins are important targets for regulating CSF production.

Based on the current findings, the TMEM67 homozygous rat is a good model of MKS as it demonstrates encephalocele-like changes, midline malformation and renal cystic disease^13–18,39,40^. In addition, the characterization of the TMEM67^+/−^ phenotype elucidates a novel and potentially useful model of more slowly progressing hydrocephalus. In the first year of life, the heterozygous animals groom themselves, reproduce normally and care for their young. However, at approximately one year of age, the animals start to exhibit signs of stress. They become unkempt in appearance and, on occasion, we have noted that the eyes seem to bulge. By 13 months of age, many of the animals have to be sacrificed for humane reasons, well before the expected life span of WT rats (~2 years). MRIs conducted to compare WT and heterozygous animals at 8 months of age, when the animals are still healthy, indicated that the hydrocephalus present at P18 is maintained chronically over the life of the TMEM67^+/−^ animals. These studies represent the first demonstration of hydrocephalus in the heterozygous Wpk rats and as such makes them unique as a slowly progressing hydrocephalic model. These studies also highlight the importance of further studies to elucidate the factors that control electrolyte transporters in the choroid plexus barrier epithelial cells and are likely to be important pharmaceutical targets in the control of CSF production.

## Methods

### Generation of a derived cleaved amplified polymorphic sequences (dCAPS) marker for the Wpk mutation

To detect WT and mutant alleles of the Wpk rat TMEM67 gene, a derived cleaved amplified polymorphic sequences (dCAPS) approach was employed^19^. We located the region containing the point mutation in the Wpk rat in chromosome 5 (RefSeq NC_005104) and TMEM67 mRNA sequences (RefSeq NM_001107916) from NCBI. The C to T substitution in the Wpk mutant^13^ occurs at nucleotide 1186 in exon 12 within the TMEM67 mRNA. The sequence containing the mutation did not lie within any known restriction site, making it impossible to design a traditional CAPS marker. Therefore, a dCAPS approach was adopted using MwoI restriction enzyme since all point mutations except A to T can be converted into a marker for MwoI.19 The Wpk/TMEM67 dCAPS forward primer was 5′-CCTGGCTGACTTTCCCAGTG-3′ and reverse primer was 5′-GTATATTCCAGGTAAATATCAGCAAACACA-3′. The 3′-end of the dCAPS forward primer was directly 5′ to the mutation site. The reverse primer was 3′ to the point mutation (‘Reverse’ in Fig. 1a) in the intron immediately following the exon containing the point mutation. The mismatched G at the 3′-end of the forward primer and the mismatched GC near the 3′-end of the reverse primer create the left- and right-hand GC dinucleotides within the MwoI site after PCR. In preliminary amplifications, the dCAPs primer pair generated several discrete PCR products with rat genomic DNA because of the presence of closely related target sequences in the genome. This complication was alleviated by using nested PCR. The forward and reverse primers for nested PCR were 5-GCTATGAGAGAGCAGGGGAG-3′ and 5′-AACTCCTGGCTGACTTTCCC-3′, respectively (Fig. 1a). Nested PCR provided a PCR product of 157 bp (Fig. 1b) to use as a template for subsequent amplification with the dCAPS primers (Fig. 1c). Total DNA for PCR analysis was isolated from tail clips using QIAamp DNA Kit (Qiagen).

Nested PCR was carried out in 20 μl containing 25 ng genomic DNA following the manufacturer’s instructions (GoTaq®Green Master Mix). Cycling conditions were: denaturation at 95 °C for 2 min, followed by 30 cycles of denaturation at 95 °C for 30 sec, annealing at 49.5 °C for 15 sec, and extension at 72 °C for 30 sec. The dCAPS PCR was performed in 20 μl reactions containing 2 μl of amplified products from the nested PCR with the following cycling conditions: 95 °C for 5 min (denaturation), followed by 30 cycles at 95 °C for 15 sec (denaturation), 44 °C for 15 sec (annealing), and 72 °C for 5 sec (extension). PCR products were digested with MwoI (New England Biolabs) in total reaction volumes of 50 μl by adding 10 μl of PCR product to 5 μl of the 10X CutSmart buffer (1:10 dilution) containing 5 units of MwoI (34 μl H2O, 10 μl PCR product, 5 μl 1X CutSmart buffer, and 1 μl MwoI). The samples were then incubated at 60 °C for 1 h. Following digestion, the samples were separated by electrophoresis on vertical 15% polyacrylamide gels in 1X Tris/boric acid/EDTA (TBE) buffer and visualized by staining with ethidium bromide. Two different size markers of 50 bp DNA ladder (New England Biolabs, NEB#B7025) and 10 bp DNA ladder (Invitrogen/ThermoFisher, SM1313) were used for the nested PCR, and the PCRs pre- or post-MwoI digestion, respectively.

### Animal procedures

The Wpk rats on a Wistar background were bred as previously described^12,13,20^. Neonatal pups (postnatal day 0 to 18; P0 to P18), both male and female were randomly assigned to be used based on genotype. For the adult rat radiological study, TMEM67^+/−^ rats at the age of 8 months were used (n = 8) as compared to the WT controls (n = 10). At the time of sacrifice, the body weights were measured and the animals were anesthetized with an overdose of sodium pentobarbital (150 mg/kg body weight). Blood was collected via cardiac puncture and the carcass was flushed with saline and perfused intracardially with 4% paraformaldehyde (PFA). Kidneys and hearts were removed and weighed. Biparietal and vertical (palate to cranial cap) head measurements were taken with Vernier calipers (Manostat, Merenschwand, Switzerland). In animals that were not perfused, the CSF was collected with 25-gauge needle through cisterna magna in a sagittal position and transferred to the freezer (−80 °C) prior to electrolyte analysis.

Rodent use and procedures conformed to the National Institutes of Health (NIH) guidelines and were approved by the Institutional Animal Care and Use Committees at Indiana University Purdue University Indianapolis and the Indiana University School of Medicine. Both male and female animals were used.

### Electrolyte analysis

CSF was collected from terminally anesthetized normal and homozygous pups aged 15–20 days. In the case of the hydrocephalic animals, each n was from an individual animal. For the normal animals, CSF was pooled from 4–6 animals for each determination. Ionic compositions of Na^+^, Cl^−^, and K^+^ were quantified and osmotic concentration [mOsM] in the CSF was determined. The electrolyte quantification was performed at the IU Health Pathology Laboratories of the Indiana University School of Medicine.

### Magnetic resonance imaging (MRI)

On day P7–8 and P17–18, rat pups were briefly removed from their litter, sedated with 5% isoflurane (balance medical oxygen) and anesthesia maintained with 1–2% isoflurane (balance medical oxygen). Adult animals (P240 ± 3 days) were lightly sedated with 2% isoflurane. High resolution T2-weighted (T2W) MRI images were acquired using a 3 T clinical MRI scanner (IMAGNETOM Trio, Siemens Healthcare, Erlangen, Germany) outfitted with a dedicated 4 channel rat head coil and bed system (RAPID MR, Columbus, OH). Images were acquired using a 3D SPACE sequence with the following acquisition parameters: (TA: 5.5 min; TR: 2080 ms; TE: 162 ms; FS: On; Ave: 2; Flip Angle: 150; Slice Thickness 0.2 mm: Matrix: 192 × 192; FOV: 35 mm × 35 mm) yielding 0.18 × 0.18 × 0.2 mm resolution images. Volumes of interest (VOI) on lateral ventricles were determined from threshold-based image segmentation of native CSF contrast, and images were quantified for lateral ventricular volumes using Analyze 12.0 (AnalyzeDirect, Overland Park, KS).

### Histology

Rats were terminally anesthetized with sodium pentobarbital and intracardial perfusion was conducted with saline followed by 4% paraformaldehyde (PFA). Harvested brains and kidneys were immersed in 30% sucrose in PBS at 4 °C for 2–3 days or when cryo-protected tissue has sunken down to the bottom of the dish. The tissue in sucrose solution was briefly washed with PBS and further placed in a square mold with optimal cutting temperature (O.C.T.) compound (Tissue-Tek) at −170 °C using dry ice and isopentane (Fisher Scientific), in a rectangular aluminum tray. Snap-frozen molds containing tissue specimen were kept at −80 °C until cryo-sectioning^25^. Following the rostral-caudal axis in the rat brain atlas^41^, 2 mm rostral to the bregma (bregma + 2.0 mm) was identified during sectioning and labeled ‘start of the lateral ventricle or SLV’. The selection of slides for comparison among genotypes was based on the distance from the SLV. 2 mm caudal to SLV corresponds to the bregma in controls (P18). In massively hydrocephalic sections, however, the volume of the brains was higher than that of controls. Therefore, serial sections were obtained prior/post the bregma target of interest to determine the identical anatomical location for the sections from control and hydrocephalic brains. Appearance of third ventricles, dorsal and ventral hippocampus, aqueduct, SCO, the fourth ventricle and cerebellum during coronal sectioning formed secondary references to the SLV in the sections from hydrocephalic brains as compared to the control. During cryosectioning, 20 μm thickness was applied at −23 °C. Selected serial sections were stained with hematoxylin alone or hematoxylin and eosin. Stained sections were imaged with a light microscope (M420, Heerbrugg, Switzerland), and images were captured using Kodak camera (DC290, Rochester, NY). In assessing CSF circulation, sterile filtered 1% Evan’s Blue (4 μl/g body weight) in PBS was injected to cisterna magna using a 25-gauge needle at about 6 μl/sec^21^. To examine barrier function within the central nervous system, 1% Evan’s Blue (4 μl/g body weight) in PBS was injected intracardially using a 25-gauge needle, prior to 4% PFA perfusion.

### Hematoxylin stain

Frozen sections were air dried at room temperature for 10 min. Sections were stained in hematoxylin solution (Surgipath 01522) for 1 min. Sections were then washed with ultrapure water or Milli-Q® (MQ) water for 4 min (twice), acidic alcohol solutions for 5 min, and MQ water for 4 min. Sections were then immersed in ammonia water (or sodium bicarbonate) 5–6 times slowly, MQ water for 4 min, and graded ethanol (EtOH) in the following order: 80% EtOH for 4 min, 95% EtOH for 1 min (three times), 100% EtOH for 1 min, 100% EtOH for 3 min, Xylene for 5 min, and mounted with Permount medium (Fisher Scientific).

### Hematoxylin and eosin stain

Sections were allowed to come to room temperature then stained with hematoxylin (Poly Scientific #s212A) for 3 minutes. Stained sections were rinsed with deionized water before washing in tap water for 5 minutes. Slides were dipped in acid ethanol (1 mL concentrated HCl + 400 mL 70% ethanol in water) 8–12 times, rinsed twice in tap water for 1 minute each, then rinsed in deionized water for 2 minutes. Sections were then stained with eosin for 30–45 seconds and dehydrated with three 5 minute washes in 95% ethanol, followed by three 5 minute washes in 100% ethanol, and finally three 15 minute washes in Xylene. Slides were then coverslipped with Permount mounting media (Fisher Scientific #SP15–100) and allowed to dry overnight.

### Immunofluorescence

Slides were incubated with primary antibodies diluted in blocking solution overnight at 4 °C, rinsed, and incubated with the secondary antibodies for 1 h at room temperature. Primary antibodies were rabbit anti-claudin 1 and anti-aquaporin 1 (1:100 dilution; Abcam), and anti-Arl13b (1:100, Proteintech). The secondary antibody was Alexa Fluor dye-conjugated goat anti-rabbit IgG (diluted 1:1000; Invitrogen). For nuclear staining 4′, 6-diamidino-2-phenylindole (DAPI) 500 ng/ml (Sigma) was used. Confocal images were taken on a Leica TCS SP8 high speed multiphoton and confocal imaging system. For low magnification fluorescence micrographs, a Nikon 90i microscope with Spot RT cooled CCD camera was used.

### Scanning electron microscopy (SEM)

The specimens were fixed with the appropriate aldehyde fixative for a minimum of 2 hours. They were then rinsed with PBS and post fixed with 1% osmium tetroxide in 0.1M Phosphate Buffer for 2 hours. Post fixation and rinsing with PBS, the specimens were dehydrated through a series of ethyl alcohols then chemically dried using HMDS (hexamethyldisilazane, Electron Microscopy Sciences, Fort Washington, PA). The schedule was as follows: 2 parts 100% ethyl alcohol/1-part HMDS for 15 minutes, 1 part 100% ethyl alcohol/2 parts HDMS for 15 minutes, then 2 changes for 15 minutes each with 100% HDMS. After HDMS depletion, the specimens were allowed to air-dry in a hood overnight, mounted on aluminum stubs with adhesive tabs, and sputter coated for 3 minutes using a Polaron (Energy Beam Sciences, Agawam, MA). The specimens were viewed on a JEOL 6390LV (Peabody, MA) scanning electron microscope and digital images were taken.

### Image analysis

To quantify Evans blue leakage in the brain, images of coronal sections were analyzed using NIH ImageJ. Briefly, micrographs showing hematoxylin or H&E stained sections were processed through line measure function. For the assay of barrier functions, photographs with Evans blue extravasation were processed through sharpen, edge detection, binarization, and pixel count. This enabled the calculation of pictorial elements or blue dye stained areas of the brain (with albumin to which Evans blue binds). Serial sections with 100 µm interval involving forebrain, midbrain, and hindbrain from three animals per genotype were used. To quantify cilia length, blinded observers who did not have information on genotypes were asked to measure the cilia length as exemplified or indicated with arrows (Fig. 4). Using ImageJ, the known scale bar (5 μm) was used as an input and the line or curve measurement function was used to quantify the length of mono-cilia in micrometer scale.

### Statistical analysis

Statistical analyses were performed using SigmaPlot (version 11, Systat Software Inc.) and Prism (GraphPad version 7). Normality of data distribution was tested using the F-test for unequal variance. Normally distributed data were analyzed using Student’s t-test and Tukey’s post-hoc test for pair-wise comparison after ANOVA when comparing two or three groups respectively. Non-normally distributed data were analyzed using the nonparametric Mann-Whitney test and Kruskal-Wallis with Dunnett’s post hoc tests when comparing two and three data groups, respectively. Data were expressed as average ± standard error of the mean (S.E.) and were considered significant at the p ≤ 0.05 level.

## Data Availability

All data generated or analyzed during this study are included in this published article.
